# Suppression of DNA/RNA and protein oxidation by dietary supplement which contains plant extracts and vitamins: a randomized, double-blind, placebo-controlled trial

**DOI:** 10.1186/s12944-018-0836-z

**Published:** 2018-08-16

**Authors:** Elizabeth Fragopoulou, Lamprini Gavriil, Chrysa Argyrou, Ioannis Malagaris, Maria Choleva, Smaragdi Antonopoulou, Georgia Afxentiou, Eleana Nikolaou

**Affiliations:** Department of Nutrition and Dietetics, Harokopio University, 70 Eleftheriou Venizelou Avenue Kallithea, 17671 Athens, Greece

**Keywords:** *Aloe vera*, Plant extracts, Protein oxidation, DNA oxidation, Vitamins, Anti-oxidants

## Abstract

**Background:**

Excessive oxidative stress may impair bio-molecules and cellular function. Multi antioxidant supplementation is thought to be more effective than a single antioxidant probably through the synergistic or complementary action of natural substances that could enhance the prospective effect.

**Methods:**

In order to estimate the effect of a plant extract based supplement in apparently healthy volunteers’ oxidative stress markers, a double-blind and placebo controlled intervention was performed. 62 apparently healthy volunteers, overweight with medium adherence to the Mediterranean diet, were recruited and randomly allocated into two intervention groups (supplement or placebo) for 8 weeks. Basic biochemical markers, oxidized LDL (oxLDL), resistance of serum in oxidation, protein carbonyls in serum and 8-isoprostane and DNA/RNA damage in urine were measured.

**Results:**

No differentiation was observed in basic biochemical markers, in oxLDL levels as well as in serum resistance against oxidation, during intervention in the examined groups. A significant resistance regarding urine isoprostanes levels in the supplement group compared to the placebo one, was observed. Reduction on DNA/RNA damage and on protein carbonyls levels (almost 30% and 20% respectively, at 8 weeks) was detected in volunteers who consumed the supplement compared to the control group.

**Conclusion:**

Consumption of plant extract based supplement seems to reduce DNA/RNA and protein oxidation and in less extent lipids peroxidation.

**Trial registration:**

ClinicalTrials.gov Identifier for this study is: NCT02837107.

## Background

The term “oxidative stress” refers to imbalance between oxidants and antioxidants in favor of the oxidants that potentially could lead to damage [1, 2]. While it is well accepted that a low level of RONS production is necessary to maintain physiological function [1], on the other hand excessive formation of RONS is believed to cause damages in biomolecules. Defense systems against RONS include endogenous/exogenous antioxidants, scavenger enzymes and chelating proteins for pro-oxidant metals [3, 4]. Damage of lipids, proteins and DNA/RNA, to cellular and tissue level, as a consequence of oxidative stress has been linked not only to a number of serious diseases, including cancer, CVD but also accelerates the process of aging [5, 6].

Diet is thought to play a major role in the regulation of oxidative stress [7]. Many epidemiologic studies have reported an inverse association between consumption of vegetables, fruits, foods rich in antioxidant compounds and the risk of chronic diseases, especially cancer and CVDs [7, 8]. Several in vivo and in vitro studies demonstrate the existence of bioactive components in fruits and vegetables, which could reduce oxidative status [9, 10]. Many vitamins could also act as anti-oxidants or could be implicated in the mechanisms of oxidative stress. However, the promising in vitro data concerning vitamins have not always been verified in vivo although many clinical trials have been conducted with vitamins (E, C or their combinations). Therefore, in vivo protective effect of vitamins and phytochemicals against the end point of several diseases remains uncertain [11–15]. Consequently, the possibility that the complex mixture of phytochemicals in foods may contribute to their protective effects has been raised [16]. In this concept, multiple compounds combination could act through complimentary or synergistic mechanisms to present a greater biological effect than the one single component.

Many natural products, especially herbs, have been investigated for their antioxidant activity. *Aloe barbadensis* miller or commonly known as *Aloe vera* has a long-standing tradition within herbal medicine. The processing of *Aloe vera* leaf pulp has become a large worldwide industry and it has been used as an ingredient in the production of functional foods and gel-containing health beverages [17]. So far in *Aloe vera* gel, over 75 different active constituents have been found and the major ones include minerals, amino acids, polyphenols and polysaccharides [17–20]. The antioxidant effect of *Aloe vera* has been reported both in vitro [21–25] and in vivo in animals [26]. In detail, vitamins A, C, E, B1 (thiamine), B3 (niacin), B2 (riboflavin) and components such as choline, folic acid, phenolic compounds and polysaccharides act directly as free radical scavengers against DPPH radical, superoxide anion, and hydrogen peroxide [17–20]. Also, it has been reported that *Aloe vera* reduces lipid peroxidation and the formation of hydroperoxides and inhibits prostaglandin E2 production from arachidonic acid probably through the ROS/NOS scavenging mechanism. Furthermore, *Aloe vera* seems to increase anti-oxidant enzymes e.g. SOD, catalase, glutathione-S-transferase. Finally, *Aloe vera* gel, specifically acemmanan and other polysaccharides, depicts immunomodulating effects that probably could modulate further oxidative stress [17–20]. On the other hand, the antioxidant effects of grapes are more studied. Grape juice enhances the anti-oxidant enzymes glutathione peroxidase and catalase activities [27], inhibits ROS production [28] and protects cells against DNA damage [28]. In specific, resveratrol, a phenolic phytochemical that is abundant in several plants such as grapes, and *Polygonum cuspidatum*, activates anti-oxidant enzymes (SOD, catalase, GPx, glutathione reductase, glutathione-S-transferase, heme oxygenase), inhibits pro-oxidant enzymes (NADPH oxidase, xanthine oxidase, myeloperoxidase) and acts as free radical scavenger [29, 30]. *Camellia sinensis* (green tea) mostly contains flavonoids and especially catechins. Catechins have been reported to exert anti-oxidant effect through activation of anti-oxidant enzymes (SOD, catalase, GPx), inhibition of pro-oxidant enzymes (NADPH oxidase, lipoxygenase, cyclooxygenase, xanthine oxidase, inducible nitric oxide synthetase) and also by acting as free radical scavengers [31, 32].

Therefore, having in mind that there are no data about the anti-oxidant potential of *Aloe vera* in humans and also that *Aloe vera* gel improves the bioavailability of co-administrated vitamins in human subjects [33], it was hypothesized that a combination of vitamins (vitamin B1, folic acid, vitamin B12 and vitamin E) and phytochemicals (Gel of *Aloe barbadensis* miller, grape juice, green tea extract and *Polygonum cuspidatum* extract that contain resveratrol) may constitute an effective tool to protect against oxidative stress. To investigate this hypothesis, a double-blind, randomized, and placebo-controlled clinical trial was conducted in order to study the effects of a multi-micronutrient supplement against oxidative stress in apparently healthy adults. Focusing on oxidative stress evaluation, lipid, protein and DNA/RNA end oxidative products, oxLDL, resistance of serum in oxidation as well as the activity of anti-oxidant enzymes were measured.

## Methods

### Supplement

The supplement (Mind Master) (patent DE 102013205049A1) and a look-alike placebo were custom prepared and donated by LR Healthy and Beauty Systems LTD. The supplement (MM) contained per 80 ml, *Aloe barbadensis* miller gel (USA/Mexico 36%), grape juice (32.5%), *Polygonum cuspidatum* extract (0.01%, that contains 10% resveratrol), green tea extract (0.4%) and vitamins. The supplement comprises plant extracts; vitamins; porhyrins and stilbenoids. In specific, the daily dosage of the supplement product (80 ml) provides vitamins /trace elements at equal or lower than Recommended Dietary Allowance (RDA): 1.1 mg vitamin B1 (100% RDA), 2.5 μg vitamin B12 (100% RDA), 12 mg vitamin E (α-ΤΕ) (100% RDA), coenzyme Q10 (0.09%), 200 μg folic acid (100% RDA), 27.5 μg selenium (50% RDA), 4.2 mg iron (30% RDA). The placebo contained *Aloe barbadensis* miller gel (USA/Mexico 3.6%), and some excipients in order to be similar in taste, appearance and color with the experimental formula.

According to ORAC measurement [34] the daily dosage of the supplement product (80 ml) provides 3440 μmol Trolox equivalents per day. In addition, total phenolic determination was performed using a modified method of Singleton and Rossi and 3.1 ± 0.09 mg gallic acid/mL in supplement and 0.3 ± 0.009 mg gallic acid/mL in placebo was estimated [35].

### Study protocol

**Fig. 1:**
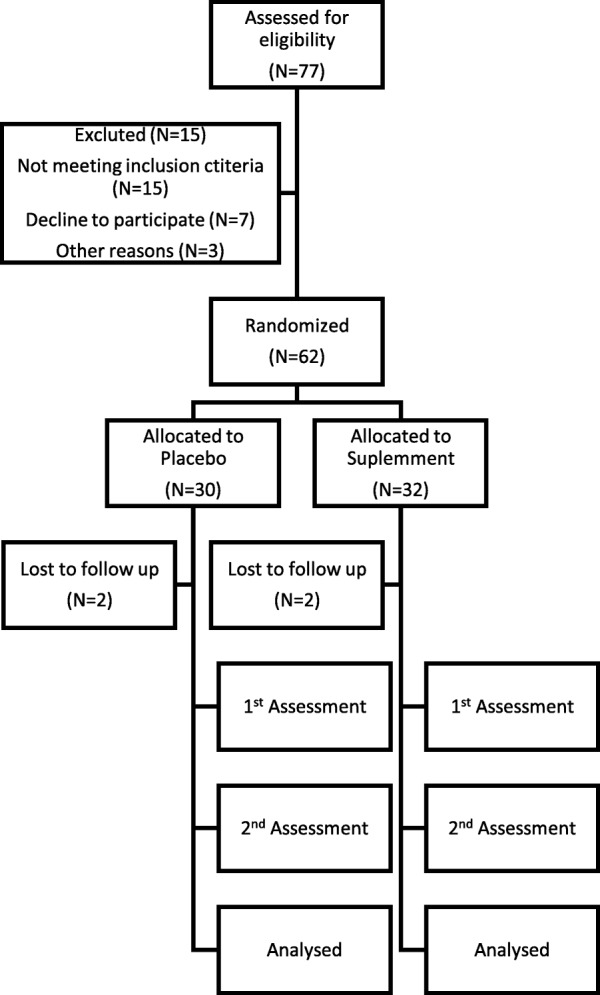
Study flow chart

This was a double-blind, block randomized, parallel-arm, placebo-controlled, eight-week study. Initially, 77 apparently healthy volunteers were recruited to participate to current study (Fig. 1). Exclusion criteria were the regular use of dietary supplements or medications, being on slimming or any other special diet, being an athlete, diagnosis of hypertension, metabolic or endocrine disease, gastrointestinal disorders, or a recent history of medical or surgical events. 62 volunteers were enrolled in the study and assigned to either the MM group (*n* = 32) or the placebo group (*n* = 30) using a stratified randomization of age, sex and Body mass index (BMI) distribution between the two groups. The randomization code was prepared by a staff member who was not involved in running the trial, by using computer-generated random numbers. 58 subjects completed the intervention (two subjects dropped out for personal reasons and the others were not consistent with the protocol). At the initiation of the study, the subjects received 5 bottles (0.5 L each) of the MM or placebo, which were made indistinguishable by their identical packaging. At 4 weeks the subjects received additionally 5 bottles. The subjects were asked to consume 80 mL per day, preferably after meals. The dose was chosen based on the commercially recommended level. At each visit, the remaining volume of the supplement was counted by research coordinators. The subjects were excluded from the analysis if they consumed < 80% of the recommended dose. The study was undertaken at the Metabolic Unit of the Department of Nutrition and Dietetics, Harokopio University.

### Anthropometric measurements

Anthropometry was carried out on each visit. Weight was measured to the nearest 0.1 kg using a digital scale and height to the nearest 0.1 cm using a stadiometer with head in horizontal Frankfurt plane. Both measurements were taken with the subject in light clothing and without shoes. BMI was then calculated as weight (kg) divided by height squared (m^2^).

### Lifestyle variables assessment

The subjects were instructed to maintain their usual pattern of dietary intake and not to consume any other supplement during the study period. At 0, 4 and 8 week of intervention, participants were interviewed about the 3 days dietary intake using the 24-h recall method. Based on 24-h recall method data, adherence to Mediterranean diet was evaluated using the MedDietScore (range 0–55, with higher values indicating greater adherence) [36]. All food intake data were analyzed for energy and macronutrient content using the Diet Analysis Pro Programme. A physical activity questionnaire was used to evaluate the level of physical activity, which was expressed as physical activity levels (PAL) [37].

### Gastrointestinal symptoms

The subjects were asked to report any possible adverse events and gastrointestinal symptoms were evaluated as a 7-d symptom score, using a daily questionnaire in which symptoms (i.e. abdominal pain, bloating, flatulence, borborygmi) were graded from 0 (no symptoms) to 4 (severe symptoms) [38]. The total weekly symptom score was calculated as the sum of these symptoms (range: 0–112). Stool frequency and consistency of evacuations by Bristol Stool Scale were also noted during this time.

### Blood sampling and handling

At the beginning of the study (0 weeks), at 4 weeks and at the end of the intervention (8 weeks), 12 h fasting venous blood was collected. All blood samples were collected from the brachial vein of the volunteers. Venous blood samples, for the isolation of serum, were drawn into evacuated glass tubes. After 45 min incubation in room temperature, serum was collected by centrifuging at 1500 x g for 10 min. For plasma isolation, blood was drawn into evacuated EDTA vacutainers, immediately centrifuged at 1500 x g for 10 min. Also participants collected a first-morning urine sample. For the determination of 8-isoprostane 0.005% *w*/*v* butylated hydroxytoluene was added in the urine sample. Plasma, serum and urine were immediately aliquoted and stored at approximately -80 °C.

### Isolation of leukocytes from heparinized blood

Five mL of heparinized blood were obtained from each volunteer. In order to induce erythrocyte sedimentation, 1.7 mL of dextran solution (3% dextran in NaCl 0.15 M) was added and the mixture was kept for 1 h at room temperature. The leukocyte rich supernatant was then centrifuged at 500×g for 10 min at room temperature. Contaminating erythrocytes of the sediment were lysed adding lysis solution consisting of 155 mM NH_4_Cl, 10 mM KHCO_3_ and 0.1 mM EDTA and then removed with a centrifugation at 300×g for 10 min at room temperature. The pelleted cells were resuspended in 1 mL of a buffer containing 50 mM Tris-HCl (pH 7.4), 0.25 M sucrose and 1 mM DTT and then sonicated on ice for 4 times of 10 s each. The leukocyte homogenate was aliquoted and stored at -80 °C. Protein concentrations of all preparations were determined according to the Bradford method with the use of BSA as protein standard [39].

### Biochemical measurements

Enzymatic methods were used to determine glucose (glucose oxidase, sensitivity 0.7 mg/dL, intra-assay coefficient of variation (CV) 2.4%), triacylglycerols (TAG) (phospho-glycerol oxidase, sensitivity 3 mg/dL, intra-assay CV 1.3%), uric acid uricase, sensitivity 0.2 mg/dL, intra-assay CV 1.1%), total cholesterol (cholesterol esterase/cholesterol oxidase, sensitivity 4 mg/dL, intra-assay CV 1.6%). High density lipoprotein (HDL) cholesterol was determined using the same procedure, after precipitation of non-HDL lipoproteins with phosphotungstic acid and low-density lipoprotein LDL (LDL) cholesterol was calculated using Friedewald formula.

### Measurement of Thiobarbituric acid reactive substances (TBARS)

TBARS levels were measured in serum using a modified colorimetric method [40]. Briefly, 0.1 mL of serum was added to 0.2 mL of phosphoric acid 0.2 Μ, 0.025 mL of butylated hydroxyl toluene 5 μΜ and 0.025 μL thiobarbituric acid 0.11 M. The mixture was incubated for 60 min at 90 °C, cooled and after the addition of 0.5 mL butanol centrifuged at 12000 x g for 10 min at 4 °C. The phase of the butanol was transferred in a 96-well plate and the absorbance was measured at 532 nm. The analysis was conducted with the use of a microplate spectrophotometer (BioTek PowerWave XS2). TBARS concentration was calculated using 1,1,3,3-tetramethoxypropane as a standard. Results are expressed as μM.

### Measurement of serum resistance to oxidation (lag time)

Serum samples were also analyzed for the ex vivo serum resistance to oxidative stress, that was induced by copper sulfate (CuSO_4_) and measured by the conjugated diene formation [41]. The analysis was conducted with the use of a microplate spectrophotometer (BioTek PowerWave XS2). Results are expressed in minutes (lag time).

### Measurement of Gpx activity

The GPx activity was measured in serum by continuous monitoring of the regeneration of reduced glutathione from oxidized glutathione upon the action of glutathione reductase and NADPH [42]. The analysis was conducted with the use of a microplate spectrophotometer (BioTek PowerWave XS2). Results are expressed as units of GPx per mL of serum (U/mL).

### Measurement of SOD activity

The total SOD activity was measured in leukocytes homogenates according to the method of McCord [43]. Units are calculated based on the formula units = % inhibition/(100 − % inhibition) and results are expressed as U/mg of protein.

### Determination of oxLDL

Plasma levels of oxLDL were measured in serum by a competitive enzyme-linked immunosorbent assay using a specific murine monoclonar antibody (4E6) according to the instructions provided by the manufacturer (Mercodia, Uppsala, Sweden). Intra- and inter-assay coefficients of variation were 6.0% and 7.0%, respectively.

### Measurement of Isoprostanes and DNA/RNA damage

Urine levels of 8-epiPGF2a were determined by means of a competitive ELISA, using a commercially available kit (Cayman Chemicals, Ann Arbor, MI). Urine 8-hydroxy-2′-deoxyguanosine, 8-hydroxyguanosine, and 8-hydroxyguanine were determined by means of a competitive ELISA, using a commercially available kit (Cayman Chemicals, Ann Arbor, MI). Urinary creatinine levels are commonly used as an index of standardization for a variety of other tests.

### Protein carbonyl determination

The accumulation of oxidized proteins was measured in serum by the content of reactive carbonyls. A protein Carbonyl assay kit (Sigma Aldrich) was used to evaluate colorimetrically-oxidized proteins in serum as described in detail by the manufacturer. Oxidized protein values obtained were normalized to the total protein concentration in the final pellet in order to consider protein loss during the washing steps.

### Statistical analysis

A priori statistical power analysis showed that the number of participants in each arm, *n* = 25, was adequate to achieve statistical power equal to 83% at 5% significance level of two-sided hypotheses that evaluated 1 standard deviation (SD) differences in isoprostane levels and DNA damage. Normality was tested using the Kolmogorov-Smirnov criterion. Normally distributed continuous variables are presented as mean values ± standard deviation, while skewed variables as median and quartiles (25th–75th). Categorical variables are presented as frequencies. For the comparisons the independent samples t-test for normally distributed variables or the Mann-Whitney of the skewed and the chi-square test for categorical variables, were used. Repeated measures ANOVA was used for the comparisons of the response curves of normally distributed biochemical markers, by testing for an intervention effect and a time x intervention interaction. For the skewed biochemical markers Mann-Whitney test was used for the comparison of MM vs Placebo at 4 and 8 weeks and Friedman’s 2-way ANOVA by ranks for testing the time effect in the same intervention. All reported *P*-values are based on two-sided hypotheses and compared to a significance level of 5%. SPSS 21 (SPSS Inc., Chicago, Il, USA) software was used for all the statistical calculations.

## Results

### Compliance to treatment, nutrient intake and physical activity

Initially, 77 apparently healthy volunteers were recruited into the trial; of these, 58 completed the trial (Fig. 1). Based on the gastrointestinal records, no adverse effects of supplements were reported during this study. Compliance was very good; the median volume of supplement returned was 6 and 7 doses for supplement and placebo, respectively.

Based on 24-h dietary recall data, MedDietScore of the individuals at baseline was calculated and was found similar in both groups (*p* = 0.7) with values 29.6 ± 5.3 and 30.1 ± 5.9 for the placebo and the MM group, respectively. Details concerning subjects’ diets based on 24-h dietary recall are presented on Table 1. The dietary intake did not differ significantly between groups at any time point regarding average calorie intake and the daily consumption of macronutrients, minerals and vitamins, with the exception of the intake of the antioxidant vitamins/phytochemicals that the supplement provided. Also, no difference was observed in the basic food groups between the two groups neither at baseline nor during the intervention.

Physical activity status of the subjects did not differ significantly between the two groups at baseline (Table 1). Also, no difference in physical activity status was observed at baseline or during the intervention.

**Table 1:** Daily energy expenditure, nutrient and food groups’ intake before intervention

	Placebo (*N* = 28)	Supplement (*N* = 30)	*p*
Total energy intake (Kcal)	2000 ± 450	1991 ± 402	0.9
Carbohydrates (g)	215.0 ± 49.2	195.3 ± 49.9	0.1
Proteins (g)	75.6 ± 22.2	77.1 ± 15.5	0.7
Lipids (g)	84.8 ± 21.6	91.0 ± 23.4	0.3
SFA (g)	26.3 ± 6.6	27.2 ± 7.3	0.6
MUFA (g)	38.1 ± 13.3	43.2 ± 13.2	0.1
PUFA (g)	11.6 ± 4.6	12.1 ± 4.3	0.6
Fiber (g)	18.2 ± 6.8	17.1 ± 8.2	0.5
Alcohol	0.58 (0.0–1.45)	0.95 (0.45–1.82)	0.1
Fruits (servings/day)	1.00 (0.04–1.86)	0.58 (0.09–1.67)	0.5
Vegetables (servings/day)	1.65 ± 1.51	2.06 ± 1.35	0.2
Dairy total fat (servings/day)	2.09 ± 1.25	2.11 ± 1.16	0.9
Dairy low fat (servings/day)	0.25(0.00–0.82)	0.33 (0.00–1.00)	0.2
Red meet (servings/day)	1.03 ± 0.88	1.11 ± 0.95	0.7
Products of red meet (servings/day)	0.94 ± 0.92	0.67 ± 0.75	0.2
Chicken/turkey (servings/day)	0.30 (0.00–0.67)	0.27 (0.00–0.59)	0.7
Fish (servings/day)	0.00 (0.00–0.67)	0.00 (0.00–0.66)	0.6
Whole grain (servings/day)	0.08(0.09–1.17)	0.67 (0.00–2.2)	0.4
Refined grain (servings/day)	5.01 ± 2.7	4.99 ± 2.38	0.9
Legumes (servings/day)	0.00 (0.00–0.67)	0.00 (0.00–0.33)	0.7
Olive oil (servings/day)	2.00 (1.75–2.00)	2.00(2.00–2.00)	0.6
Junk Food (servings/day)	0.33 (0.00–0.62)	0.33 (0.00–0.67)	0.8
Physical Activity Levels	1.50 ± 0.19	1.43 ± 0.18	0.2

Intake levels were estimated from 3 days dietary intake using the 24-h recall method. Data are presented as means ± SD for normally distributed variables and as median (lower-upper quartile (25th -75th) for skewed variables t-test or Mann-Whitney test was used for the comparisons respectively

#### Participant characteristics at baseline

The subjects’ anthropometric, biochemical and haematological data before supplementation did not differ between the placebo and the supplement group (Table 2). The subjects’ values were within normal range with the exception of BMI that indicates an overweight population. In addition, no difference was observed in baseline values of oxidative stress markers between groups with the exception of protein carbonyl levels (Table 3).

**Table 2:** Basic clinical and anthropometric characteristics of participants before intervention

	Placebo (*N* = 28)	Supplement (*N* = 30)	p
Age (years)	32.9 ± 5.6	34.9 ± 5.8	0.2
Gender, (%male)	64.3	73.3	0.5
Body mass index (kg/m2)	26.6 ± 2.9	26.0 ± 1.9	0.6
Current smokers (%)	75.0	53.3	0.1
Systolic blood pressure (mm Hg)	126.9 ± 10.2	122.7 ± 10.4	0.08
Diastolic blood pressure (mm Hg)	78.5 ± 8.8	74.4 ± 8.5	0.1
WBC (10^3^/μL)	5.7 ± 1.4	6.24 ± 1.3	0.2
RBC (10^6^/μL	4.4 ± 1.4	4.54 ± 1.3	0.7
PLT 10^3^/μL	224.0 ± 59.1	240.64 ± 32.4	0.2
Hb (g/L)	14.6 ± 2.7	15.14 ± 1.3	0.5
Ht (%)	40.9 ± 4.2	40.34 ± 4.0	0.5
Glucose (mmol/L)	5.31 ± 0.56	5.62 ± 0.71	0.08
Cholesterol (mmol/L)	4.85 ± 1.11	5.20 ± 1.09	0.2
HDL-cholesterol (mmol/L)	1.27 ± 0.36	1.19 ± 0.4	0.4
LDL-cholesterol (mmol/L)	3.20 ± 1.13	3.57 ± 1.13	0.2
Triacylglycerols (mmol/L)	0.82 ± 0.44	1.04 ± 0.73	0.2
Uric acid (mmol/L)	0.28 ± 0.07	0.27 ± 0.07	0.6

Data are presented as means ± SD for normally distributed variables or as relative frequencies (%) for categorical variables t-test or chi-square was used for the comparisons respectively

**Table 3:** Levels of oxidative stress markers of participants before intervention

	Placebo (*N* = 28)	Supplement (*N* = 30)	*p*
LagTime (min)	56.0 ± 20.4	53.0 ± 12.6	0.5
Isoprostane(ng/mmol creatinine)	75.1 (47.5—261.1)	96.1 (52.1–236.7)	0.4
TBARS (μM)	5.21 ± 1.22	5.05 ± 1.59	0.6
oxLDL (μg/mL)	1.23 (0.681–2.35)	1.54 (1.01–2.31)	0.1
8-hydroxy-2΄- deoxyguanosine (μg/mmol creatinine)	10.5 (5.23–21.4)	12.0 (8.36–16.5)	0.9
Protein Carbonyls (nmol/mg protein	81.1 (61.3–93.7)	95.3 (70.6–133.1)	0.04
Gpx activity (U/mL)	0.0826 (0.0734–0.0964)	0.0911 (0.0774–0.0911)	0.4
SOD activity (U/mg)	16.8 (9.64–27.1)	18.2 (11.2–25.5)	0.6

Data are presented as means ± SD for normally distributed variables and as median (lower-upper quartile (25th -75th) for skewed variables, t-test or Mann-Whitney test was used for the comparisons respectively

### Basic biochemical markers

No difference was observed in glucose, total cholesterol, HDL-cholesterol, LDL-cholesterol, TAG and uric acid levels in any group during the intervention (Table 4).

**Table 4:** Levels of basic anthropometric and biochemical markers during intervention

Time (weeks)		0	4	8	p_trial_ p_time_ p_trial *_ p_time_
BMI (kg/m2)	Placebo (*N* = 28)	26.6 ± 2.9	26.7 ± 3.0	26.7 ± 3.0	0.7 0.7 0.2
	Supplement (*N* = 30)	27.0 ± 2.0	26.8 ± 2.0	26.8 ± 1.9	
Systolic blood pressure (mm Hg)	Placebo (*N* = 28)	78.5 ± 8.8	74.0 ± 8.8	72.4 ± 8.2	0.8 0.1 0.1
	Supplement (*N* = 30)	74.5 ± 8.5	75.3 ± 8.6	73.3 ± 8.4	
Diastolic blood pressure (mm Hg)	Placebo (*N* = 28)	126.9 ± 10.2	120.2 ± 12.7	119.2 ± 9.7	0.8 0.1 0.2
	Supplement (*N* = 30)	122.7 ± 10.5	117.4 ± 10.4	120.0 ± 7.4	
Glucose (mmol/L)	Placebo (*N* = 28)	5.31 ± 0.56	5,27 ± 0.54	5.32 ± 0.63	0.09 0.3 0.1
	Supplement (*N* = 30)	5.62 ± 0.71	5.63 ± 0.73	5.46 ± 0.69	
Cholesterol (mmol/L)	Placebo (*N* = 28)	4.85 ± 1.11	4.71 ± 0.98	4.69 ± 1.07	0.1 0.06 0.9
	Supplement (*N* = 30)	5.20 ± 1.09	5.10 ± 1.04	5.07 ± 1.11	
HDL-chol (mmol/L)	Placebo (*N* = 28)	1.27 ± 0.37	1.23 ± 0.35	1.25 ± 0.35	0.4 0.3 0.6
	Supplement (*N* = 30)	1.19 ± 0,41	1.17 ± 0.37	1.16 ± 0.37	
LDL-chol (mmol/L)	Placebo (*N* = 28)	3.20 ± 1.13	3.12 ± 1.06	3.06 ± 1.17	0.1 0.2 0.9
	Supplement (*N* = 30)	3.58 ± 1.18	3.53 ± 1.16	3.49 ± 1.18	
Triacylglycerols (mmol/L)	Placebo (*N* = 28)	0.82 ± 0.44	0.83 ± 0.54	0.81 ± 0.46	0.1 0.6 0.1
	Supplement (*N* = 30)	1.04 ± 0.73	0.97 ± 0.68	1.06 ± 0.78	
Uric acid (mmol/L)	Placebo (*N* = 28)	0.28 ± 0.07	0.27 ± 0.07	0.28 ± 0.07	0.5 0.3 0.2
	Supplement (*N* = 30)	0.27 ± 0.07	0.27 ± 0.06	0.27 ± 0.07	

Data are presented as means ± SD for normally distributed variables. Repeated measures ANOVA was used for the comparisons

### Lipid oxidation biomarkers

To assess lipid oxidative damage, lipid peroxidation products, by determining the levels of TBARS, oxLDL, isoprostanes as well as the resistance of serum to oxidation, were measured (Table 5). There was no significant overall change in TBARS (ptrial = 0.8, ptime = 0.5, ptime x trial =0.2), oxLDL levels and in the resistance of serum to oxidation (LagTime) (ptrial = 0.8, ptime =0.5, ptime x trial =0.3). A significant resistance against oxidative damage in the supplement group compared to the placebo group regarding urine isoprostane levels was observed. In detail, 2.7% increase in placebo versus 9.7% decrease in supplement group at 4 weeks (*p* = 0.05) and 29.8% increase in placebo versus 21% decrease in supplement group at 8 weeks (*p* = 0.003) was revealed.

**Table 5:** % change of oxidative stress markers during intervention

	Placebo (*N* = 28)		Supplement (*N* = 30)		P_4–4_^b^ P_8–8_	P_Pl_^c^ P_Sup_
% of baseline levels	4 week	8 week	4 week	8 week		
LagTime (min)^a^	99.9 ± 17.2	98.9 ± 12.4	96.1 ± 17.1	102.2 ± 19.8		
Isoprostane (ng/mmol)	102.7 (83.5–136.1)	129.8^#^ (100.2–186.9)	90.3 (58.3–112.1)	79.0 (45.6–122.9)	0.05 0.003	0.005 0.3
oxLDL (μg/mL)	97.1 (89.0 0–104.4)	100.5 (87.7–110.0)	100.3 (91.9–112.5)	101.7 (89.2–112.1)	0.5 0.1	0.2 0.8
TBARS (μΜ)^a^	107.1 ± 27.4	100.3 ± 25.5	100.0 ± 26.7	104.3 ± 31.8		
8-hydroxy-2΄-deoxyguanosine (μg/mmol)	96.1 (83.8–128.8)	102.8 (88.9–145.2)	85.2^,#^ (60.9–101.8)	72.3^#^ (45.2–102.6)	0.02 0.000	0.4 0.003
Protein Carbonyls (nmol/mg protein)	112.8 (91.6–120.5)	104.5 (91.5–118.1)	83.3^#^ (71.7–102.3)	81.3^#^ (61.7–100.6)	0.000 0.001	0.3 0.003
Gpx activity (U/mL)	95.8 (87.6–116.7)	94.7 (74.8–115.4)	100.0 (87.6–115.3)	102.9 (88.2–117.5)	0.8 0.1	0.3 0.9
SOD activity (U/mg protein)	106.4 (75.0–160.4)	88.1 (66.7–151).7	63.6^#^ |(42.8–127.4)	71.3 (50.4–126.8)	0.01 0.2	0.7 0.008

Data are presented as means ± SD for normally distributed variables and as median (lower-upper quartile (25th -75th) for skewed variables

^a^For normally distributed variables repeated measures ANOVA was used for the comparisons (All p’s > 0.05, result section)

^b^For skewed variables Mann-Whitney two independent test was used for the comparison of Supplement vs Palcebo at 4 or 8 weeks

^c^Friedman’s 2-way ANOVA by ranks was used for the estimation of time effect in placebo or Supplement trial, ^#^*P* < 0.05 pairwise comparison vs 0 week of Friedman’s 2-way ANOVA by ranks

#### DNA/RNA oxidation

In order to evaluate the measurement of DNA/RNA oxidative damage, three oxidized guanine species 8-hydroxy-2’deoxyguanosine from DNA, 8-hydroxyguanosine from RNA and 8-hydroxyguanine from either DNA or RNA were measured. A significant reduction of DNA/RNA oxidative damage in the supplement group compared to the placebo group was observed at 4 weeks of intervention (3.9% decrease in placebo versus 14.8% decrease in supplement, *p* = 0.02) which was more pronounced at 8 weeks (2.8% increase in placebo versus 27.7% decrease in supplement, *p* < 0.000). In addition, a time effect was observed only in supplement group (*p* = 0.004 at 4 weeks compared to baseline, *p* = 0.02 at 8 weeks compared to baseline).

### Protein carbonyls

Reactive species produced directly or indirectly through lipid peroxidation intermediates may also modify proteins. In order to evaluate protein oxidation, serum protein carbonyls were measured. A significant reduction of protein oxidative damage in the supplement group compared to the placebo group was observed at 4 weeks of intervention (12.8% increase in placebo versus 16.7% decrease in supplement group, p < 0.000) and at 8 weeks (4.5% increase in placebo versus 18.7% decrease in supplement group, *p* = 0.001). In addition, a time effect was observed only in supplement group (*p* = 0.003 at 4 weeks compared to baseline, *p* = 0.03 at 8 weeks compared to baseline).

#### Anti-oxidant enzymes

Finally, activities of antioxidant enzymes specifically in serum and GPx and SOD in leukocytes were also measured. Only SOD activity was significantly affected by supplementation at 4 weeks (6.4% increase in placebo versus 36.4% decrease in supplement group, *p* = 0.01) while at 8 weeks, although a difference was observed, no statistical result was reached (11.9% decrease in placebo versus 28.7% decrease in supplement group at 8 weeks with *p* = 0.2). In addition, a time effect was observed only in supplement group (*p* = 0.007 at 4 weeks compared to baseline, *p* = 0.1 at 8 weeks compared to baseline).

## Discussion

The main finding of this study is that the consumption of a multi-micronutrient supplement that provides vitamins, minerals and phytochemicals, could be effective in preserving production of isoprostanes and protecting DNA/RNA and protein against oxidative damage.

Oxidative stress along with inflammation and thrombosis play central role in many pathological situations such as atherosclerosis. Hence, antioxidant supplementation, mainly vitamin E and C, has been investigated. In this context, many descriptive studies suggest that antioxidant vitamin consumption reduces the CVD risk [11–15]. However, major randomized clinical trials revealed disappointing results and a recent meta-analysis concluded that high dose of vitamin E supplementation results in increased mortality [44]. In addition, although many studies investigating the effect of vitamins’ supplementation on the primary prevention of CVD have been conducted, the results are contradictory [45].

Even though a well balanced diet should be the first option, in some cases a need for nutritional supplements that could provide a well-balanced array of antioxidant nutrients, at relatively low dosages based on recommended dietary allowance (RDA) has been reported. Taking under consideration, that a well balanced diet includes a variety of micro-constituents, the hypothesis that antioxidant combination could be more effective than a single one, wins ever more ground. This lie in the possibility of synergistic or complementary action among the various antioxidants present in natural extracts that create a magnified effect.

Bearing in mind the previous hypothesis, a supplement that contains vitamins and plant extracts, mainly *Aloe vera* gel and also grape juice, green tea and *Polygonum cuspidatum*, could serve as a multi-functional source for a variety of chemically and functionally distinct antioxidants in a complex matrix. The function of vitamins B occurs in mitochondrial reactions and energy metabolism. Thiamin (B1) prevents lipid peroxidation and its deficiency leads to mitochondrial toxicity and oxidative stress [46]. Concerning folate, recent data suggest that its reduced derivates could interact with the endothelial NO synthase and thus influence NO bioavailability and peroxynitrite formation [47]. The *Aloe vera* gel contains several components such as chromones, anthraquinones, polysaccharides, vitamins, enzymes, and low molecular weight substances, such as organic acids and minerals that have been shown to exert potent biological activities including antioxidant properties [20, 25, 48]. In addition, data also support that *Aloe vera* results in slower vitamins’ absorption and increases the bioavailability of co-administrated vitamins E and C in human subjects [33]. However, few human clinical trials were conducted in order to study the effects of the *Aloe vera* consumption [49, 50] or supplements that combine vitamins and plant extracts [51, 52], on oxidative stress markers. Concerning tea extract, it is reported to have a potential anti-oxidant effect in humans and specifically [53, 54] green tea extract supplementation improves antioxidant status of male students after 4-weeks [55] and increases glutathione peroxidase and catalase activities in male non-smoking subjects after 6-month consumption [56].

Therefore, the aim of the present study was to investigate whether the consumption of a supplement that combines natural extracts along with vitamins in doses similar to recommended intake values would influence the oxidative stress markers in a double-blind, placebo controlled, intervention study in apparently healthy volunteers. The tested supplement contains 36% *Aloe vera* gel and provides 248 mg gallic acid equivalents and 3440 μmol Trolox equivalents per day. The volunteers that participated in the study were mainly overweight. Moreover, they were characterized by a medium level of adherence in Mediterranean diet based on the values of MedDietScore in accordance with the data from the general Greek population [57]. Generally, it has been suggested that studies of antioxidant supplements should include subjects with a low intake or poor status of antioxidant nutrients, to increase the likelihood of detecting an impact of the intervention. Based on food group analysis, the consumption of vegetables and fruits by participants in this study was low.

Many oxidative markers have been developed to evaluate the redox status in humans. Among them, the most commonly used are macromolecules’ end oxidation products. In the present study, the levels of thiobarbituric acid-reacting substances (TBARS), oxLDL as well as urinary isoprostanes were measured as end products of lipid peroxidation. No difference was detected regarding TBARS and oxLDL concentration between the two groups throughout the eight-week study period. However, previous report indicates a reduction in liver malondialdehyde formation in mice after *Aloe vera* administration [58]. On the other hand, isoprostane levels seem to be altered during the study period, and more specific, volunteers consuming the supplement revealed resistance against lipid peroxidation in comparison to those ones to the placebo group. This is consistent with a previous study that revealed that an *Aloe vera* extract had a significant reduction in the F2-isoprostanes compared to placebo after 8 week supplementation [50]. An increase was observed in isoprostane levels in the placebo group but it is difficult to ensure if the placebo composition is responsible for this effect since placebo contained *Aloe barbadensis* miller gel 3.6% vs 36% of the dietary supplement and some excipients in order to be similar in taste, appearance and color with the experimental formula. In addition, the serum resistance on ex vivo oxidation in the presence of Cu did not differ in any group or between groups during the intervention period. In contrast, a recent study observed that daily consumption of higher amount of *Aloe vera* extract (250 mL) increased total antioxidant capacity of serum after 14 days [49]. Concerning other plant extracts, the consumption of green tea extract in capsules, reduced the LDL ability against oxidation in healthy men [59] while the consumption of tea did not affect urinary 8-isoprostane in a healthy population [60]. In women, the consumption of grape powder, rich in anthocyanins, quercetin, myricetin, kaempferol, and resveratrol, reduced the urinary F2-isoprostanes after 4 weeks [61]. Therefore, regarding our results, a mild protection against lipid peroxidation was achieved after the supplementation since only one of the four lipid peroxidation markers was altered.

Accumulating evidence suggests that DNA oxidative damage plays an important role in some chronic degenerative diseases. The DNA attack by hydroxyl radical generates a huge range of base and sugar modification products. Measurement of 8-hydroxy-2' -deoxyguanosine in urine has been used to assess rates of ‘whole-body’ oxidative DNA damage. In the present study, three oxidized guanine species from DNA and RNA were determined in urine and a significant reduction in supplementation group was observed, approximately of 15%, at 4 weeks and approximately 28% after 8 weeks of supplementation. This is in line with a previous report where polysaccharide fraction obtained from *Aloe vera* decreased in vitro oxidative DNA damage [62]. As far as other plant extracts are concerned, the consumption of Korean red ginseng reduced DNA oxidation in healthy volunteers after the 8-week supplementation [63]. In addition, the consumption of tea (containing approximately 250 mg of total catechins) reduced oxidative-induced DNA damage in lymphocytes in healthy volunteers [64] and the consumption of green tea polyphenols (500 mg daily) reduced urinary 8-OHdG concentrations in postmenopausal women with osteopenia [53].

Protein carbonylation is one of the most harmful irreversible oxidative protein modifications and is considered as a major marker of oxidative stress-related disorders. In the present study, a significant reduction of protein carbonyl levels, approximately of 20%, at 4 and 8 weeks, in supplementation group compared to placebo, was observed. It should be noticed that the presentence of smokers was higher, although no significant, in the placebo group but it had lower baseline levels of protein carbonyls. However, no safe conclusion could be made concerning the interplay among smoking, levels of protein carbonyls and the observed effect since smoker’s and not smoker’s number per group is too small for a subgroup analysis. No reports concerning the effect of *Aloe vera* on protein oxidation exist. However, the daily consumption of vitamin C alone or in combination with α-tocopherol and folic acid for 10–15 weeks had no effect on protein carbonyl levels [65, 66] with the exception of subjects with low baseline ascorbate levels where a reduction was observed [66]. In addition, the consumption of green tea plus vitamin E reduced the plasma protein carbonyls in healthy elderly individuals after 12 weeks [67]. Moreover, the consumption of an antioxidant-rich concentrate of berries reduced protein carbonyls in healthy volunteers at postprandial state [68]. The consumption of a *Lemon verbena* extract reduced protein carbonyl levels in young subjects at an aerobic training routine [69]. In contrast, the consumption of beetroot juice did not affect exercise-induced oxidative stress including protein carbonyls [70].

Crucial players in anti-oxidant defense are the antioxidant enzymes such as SOD, CAT, and GPx. Superoxide dismutase catalyzes the formation of hydrogen peroxide from the superoxide radical and glutathione peroxidase catalyzes the reduction of lipid hydroperoxides to their corresponding alcohols and of free hydrogen peroxide to water. In response to supplementation, no changes in the specific activity of serum GPx were found. In contrast, leukocyte superoxide dismutase activity decreased significantly after 4 weeks of supplementation compared to baseline and placebo group. The observed decrease in leukocyte superoxide dismutase activity cannot be easily interpreted. It could be assumed that either the multi-nutrient supplementation acts as a direct scavenger of RONS and decreases the body’s need for certain antioxidant enzymes or may suppress its synthesis or shorten its half-life.

Among the limitations of the study is the fact that the participants were apparently healthy volunteers; therefore conclusions cannot be extrapolated to other populations meaning patients with several pathological conditions. Also, participants had medium adherence to a healthy dietary pattern (Mediterranean diet) and the results cannot be extrapolated to a population with optimal dietary intake. Furthermore, the evaluation of participants’ compliance to the study protocol was indirectly estimated by the measurement of the supplement’s volume that has not been consumed and not by measuring vitamins or other nutrients. Finally, it is not possible to know which component is responsible for the observed effect. Probably the effect is due to a synergistic action of the natural extracts and the vitamins.

## Conclusion

Overall, the data from the present study supports the idea that supplementation with a combination of natural extracts and vitamins significantly reduces in vivo oxidant damage of water soluble systems and in less extent the lipid-soluble ones.

## Abbreviations

BMI: Body mass index
CAT: Catalase
CVDs: Cardiovascular diseases
FFQ: Food-frequency questionnaire
GPx: Glutathione peroxidase
HDL: High density lipoprotein
LDL: Low density lipoprotein
oxLDL: Oxidized low density lipoprotein
PAL: Physical activity levels
RONS: Reactive oxygen/nitrogen species
SOD: Superoxide dismutase
TAG: Triacylglycerols
TBARS: Thiobarbituric acid reactive substances
